# Addressing researcher fraud: retrospective, real-time, and preventive strategies–including legal points and data management that prevents fraud

**DOI:** 10.3389/frma.2024.1397649

**Published:** 2024-06-27

**Authors:** James E. Kennedy

**Affiliations:** School of Philosophy, Psychology and Language Sciences, University of Edinburgh, Edinburgh, United Kingdom

**Keywords:** scientific fraud, research misconduct, data management, research quality control, research audit, regulations for science, Office of Research Integrity, defamation in science

## Abstract

Researcher fraud is often easy and enticing in academic research, with little risk of detection. Cases of extensive fraud continue to occur. The amount of fraud that goes undetected is unknown and may be substantial. Three strategies for addressing researcher fraud are (a) retrospective investigations after allegations of fraud have been made, (b) sting operations that provide conclusive evidence of fraud as it occurs, and (c) data management practices that prevent the occurrence of fraud. Institutional and regulatory efforts to address researcher fraud have focused almost exclusively on the retrospective strategy. The retrospective approach is subject to controversy due to the limitations of *post-hoc* evidence in science, the difficulty in establishing who actually committed the fraud in some cases, the application of a legal standard of evidence that is much lower than the usual standards of evidence in science, and the lack of legal expertise by scientists investigating fraud. The retrospective strategy may be reliably effective primarily in cases of extensive, careless fraud. Sting operations can overcome these limitations and controversies, but are not feasible in many situations. Data management practices that are effective at preventing researcher fraud and unintentional errors are well-established in clinical trials regulated by government agencies, but appear to be largely unknown or unimplemented in most academic research. Established data management practices include: archiving secure copies of the raw data, audit trails, restricted access to the data and data collection processes, software validation, quality control checks, blinding, preregistration of data processing and analysis programs, and research audits that directly address fraud. Current discussions about data management in academic research focus on sharing data with little attention to practices that prevent intentional and unintentional errors. A designation or badge such as *error-controlled data management* could be established to indicate research that was conducted with data management practices that effectively address intentional and unintentional errors.

## 1 Introduction

Researcher fraud is an unpleasant but necessary topic in science. Those who have investigated scientific fraud consistently believe that the amount of undetected fraud is probably much greater than the amount of detected fraud (Broad and Wade, 1982; Stroebe et al., 2012; Nelson et al., 2018). Replication and peer review are now known to be generally ineffective at detecting or deterring researcher fraud (Broad and Wade, 1982; Stroebe et al., 2012; Ritchie, 2020). Researcher fraud was a major factor motivating the replication crisis in psychology (Pashler and Wagenmakers, 2012; Stroebe et al., 2012; Nelson et al., 2018). However, it has been the least discussed and least effectively addressed factor.

After reviewing cases of scientific fraud, Stroebe et al. (2012) concluded among other things that:

- “Scientists do not expect their colleagues to falsify their data and therefore do not look for signs of fraud when reading manuscripts or articles” (p. 680);
- “Any trust-based system, as science is, is open to exploitation” (p. 683);
- “As Stapel … remarked in his letter of self-justification, scientific fraud is too easy, because there are too few control mechanisms in science. People are tempted to commit fraud when the expected rewards are great and punishment is unlikely because the risk of discovery is small” (p. 681);
- “Even if there is no doubt that fraud has occurred, it is often difficult in research published by multiple authors to identify the person or persons responsible for the fraud” (p. 680).

The adverse consequences of researcher fraud are substantial. Fraud results in invalid scientific findings and wasted resources. For example, a biomedical researcher received about three million dollars in research funding based on fraudulent claims (Miller, 2012). Similarly, a case of extensive fraud in physics resulted in millions of dollars wasted pursuing invalid claims (Reich, 2009). For research with health implications, fraud can result in direct adverse effects for human health (Steen, 2011). Cases of detected fraud not only damage the reputation of the fraudulent researcher, but also the reputations of the associated institution, coauthors, graduate students, and area of research, as well as the increasingly important credibility of science in general (Keener et al., 2023). The invalid findings remain persistent even for detected fraud. An article can be retracted, but making corrections and qualifications to all the publications that referenced the article is not feasible. Gross (2016) noted that a reported minimum time of 10 months to conduct an investigation may be an underestimate and cases taking several years have been reported. This diverts researchers and associated resources from more productive scientific work. In addition, if lawyers become involved, the legal fees can be tens of thousands to hundreds of thousands of dollars (as described in cases below).

The assumption by most scientists that their colleagues can be trusted creates an environment that often makes fraud easy and tempting, and allows fraudulent researchers to function largely unimpeded (Stroebe et al., 2012; also consistent with my research experience). Those who have looked into the problem of scientific fraud have consistently concluded that changes are needed in the research culture (Broad and Wade, 1982; Stroebe et al., 2012; Ritchie, 2020; Keener et al., 2023).

Significantly increased attention to retraction of published papers had occurred (Oransky, 2022). However, the increasing rates of retraction provide little optimism that the underlying rates of scientific fraud are decreasing. And, the amount of fraud that is not detected remains a matter of speculation. The efforts to address researcher fraud have primarily focused on retraction after evidence of fraud is found.

The present article discusses the strengths and weaknesses of three strategies for addressing researcher fraud.

1. The first strategy is the common practice of retrospective investigations after allegations or suspicions of fraud have been raised.
2. The second strategy is to obtain conclusive evidence of fraud as it occurs. This requires a sting operation.
3. The third strategy is to implement research practices that prevent opportunities for fraud. Data management practices that achieve this goal are well-established in clinical trials regulated by the U.S. Food and Drug Administration (FDA) and corresponding agencies in other countries.

It may be relevant to mention that I have direct personal experience applying all three strategies (see About the Author below). And, my experience has been that research environments that have preventive measures are highly preferable to environments that make fraud easy and tempting and then rely on efforts to obtain evidence of fraud.

## 2 The retrospective approach to fraud

The most common strategy by far for handling research fraud has been to conduct a retrospective investigation after someone raises suspicions or allegations of possible fraud. Most discussions of research fraud focus exclusively on this strategy. Regulatory programs have been developed in the U.S. that apply this retrospective strategy.

### 2.1 Science meets law

Cases of extensive scientific fraud in the 1980's resulted in political recognition that government action was needed for research funded with tax-payer dollars (ORI, (c); Gross, 2016). This recognition implied that the scientific community was not adequately self-policing. An executive order was issued in 2000 that U.S. federal agencies that fund research must have regulatory programs for addressing research misconduct (Executive Office of the President, 2000). Research institutions that receive research funds from U.S. government agencies must have written policies in place that implement the required regulatory programs.

The misconduct regulations developed by the Office of Research Integrity (ORI) in the U.S. Department of Health and Human Services Public Health Service (which includes NIH) are some of the most detailed and widely applied regulations (ORI, 2005). The ORI regulations are discussed here without repeating this citation. Also, note that reading the preamble to the regulations in the Federal Register is useful in understanding the regulations.

With the advent of regulatory programs, researcher fraud involves legal matters as well as scientific matters. An investigation of suspected fraud is guided as much or more by legal factors as by scientific factors. Researchers accused of fraud may be surprised that some regulatory requirements seem to favor the accusers. Those making allegations of fraud may be surprised by lawsuits claiming defamation.

The executive order and ORI regulations define research misconduct as “fabrication, falsification, or plagiarism in proposing, performing, or reviewing research, or in reporting research results.” Research fraud as discussed in the present paper is the falsification and fabrication components of research misconduct. Falsification and fabrication produce false scientific findings that affect the integrity of science, whereas plagiarism pertains to who gets credit for ideas. With this definition, falsification and fabrication are applicable to virtually any aspect of the research process.

The regulations specify procedures that a research institution must carry out in response to allegations of misconduct. This may include sequestering relevant records and forming a committee that conducts an investigation. The regulations also specify that whistleblowers and witnesses should be protected from retaliation. At the time this paper is being written (May, 2024), the ORI regulations are being revised (ORI, 2023). The definition of misconduct will probably not change, but the required procedures may change.

The degree of evidence required to establish that research misconduct occurred is set by legal standards and is much lower than the typical standards for evidence in science. The legal standard specified in the executive order and regulations is *preponderance of the evidence*, which means the overall evidence at least slightly favors that misconduct occurred. This is the lowest level of evidence for court cases in the U.S. legal system. A higher standard of evidence in the U.S. legal system is *clear and convincing evidence*, and the highest standard is *beyond a reasonable doubt*. The executive order explicitly rejected using clear and convincing evidence. The justification included that “While much is at stake for a researcher accused of research misconduct, even more is at stake for the public when a researcher commits research misconduct” (Executive Office of the President, 2000, p. 76262).

This low standard of evidence is favorable to the accuser, whereas a higher standard of evidence would require stronger evidence against the accused researcher. For comparison, the standards of evidence for supporting a scientific hypothesis are much closer to clear and convincing evidence and beyond a reasonable doubt than to preponderance of the evidence. The usual standards of evidence for science would be more favorable for the accused researcher.

Another bias in favor of the accusers is that the ORI regulations require that the accusers (“complainants”) and other witnesses be “interviewed” by the investigation committee, but do not provide for the accused researcher to cross examine the accusers and other witnesses. Cross examination of witnesses is a fundamental part of legal due process and is a constitutional requirement for criminal cases, but is not an absolute requirement for civil or administrative cases like research misconduct. The misconduct regulations focus on restricting information about witnesses in order to prevent retaliation against whistleblowers. This is a valid concern, but it also deprives accused researchers of a basic right in defending themselves.

Protection of whistleblowers presents a dilemma that has no good solution. An accused researcher is given an opportunity to respond to the claims by the accusers and other witnesses, but is not provided an opportunity to directly ask them questions that could bring out weaknesses in their claims. Once the process becomes more legal than scientific, other legal actions may not be surprising, such as lawsuits for defamation.

An additional conflict between regulatory and scientific perspectives is that the ORI regulations allow institutions to keep private the evidence and final report of a misconduct investigation. The institutions argue that this is needed for confidentiality of those involved. However, the secrecy also minimizes negative publicity and scrutiny for the institutions.

From a scientific perspective, an argument can be made that allegations of fraud do not turn off the basic principles of open transparent science, particularly for research funded with public money. With this argument, the evidence developed in an investigation of possible fraud should be publicly available like any other scientific evidence. Scientists must make decisions about whether to invest time and funds in further research. The fact that fraud may have occurred is not necessarily evidence that the scientific hypothesis is false. Similarly, the fact that a specific person was not found guilty of fraud does not mean that the scientific findings were valid. Scientists need to be able to evaluate the evidence in the investigation. These and related arguments are discussed in comments that were submitted about the proposed revisions to the ORI regulations (Kennedy, 2023c).

The present paper discusses legal matters in the U.S., but does not attempt to address legal matters in other countries. Topics such as the differences between scientific and legal standards for evidence may be relevant for other countries, even though the specific standards and procedures may be different.

### 2.2 Initial allegations of fraud

The process for addressing researcher fraud with the retrospective strategy begins when someone makes allegations of fraud. Whistleblowing by someone close to the research has been the most common source for initial allegations (Stroebe et al., 2012; Gross, 2016). Unfortunately, as Gross (2016, p. 705) noted (with many references) “Even if the whistle-blowing turns out to be justified, the consequences for the whistle-blowers are often disastrous in terms of their income, research, personal relations at work, and future in science, as has been repeatedly related.” Gross (2016) described the policies intended to protect whistleblowers as often of little value in practice. Hopefully, protections for whistleblowers are improving.

At the same time, whistleblowing can be abused, including unwarranted attacks motivated by interpersonal problems. This possibility must be considered in responding to the allegations. Gross (2016, p. 705) noted that “premature, inadequately justified, unjustifiable, and/or inappropriately carried out whistle-blowing can be a disaster for all involved.”

Those contemplating making allegations of fraud should (a) be reasonably confident of their claims, (b) make sure their motivations are appropriate, and (c) fully understand the applicable policies and potential consequences and risks. Every situation is different. The preferable practice would be for multiple people to express similar concerns.

Initial allegations of fraud are increasingly made by people who are distant from the research. The allegations are based on anomalies in the data that could have been an artifact or side-effect of fraud. The anomalies are discovered when data or research reports are examined carefully by independent analysts. Those who devote significant time to such investigations have become known as *data sleuths* or *science sleuths*. Notably, Elisabeth Bik became a full-time science sleuth in 2019 and has been involved in many high-profile findings of scientific fraud, including thousands of falsified images (Shen, 2020; Balthazar, 2024; Bik, 2024).

Simonsohn, Simmons, and Nelson have become prominent in making allegations of fraud based on data anomalies in human behavior research (Simonsohn, 2013, 2019; Nelson and Simonsohn, 2014; Yu et al., 2018; Simonsohn et al., 2021, 2023a,b,c,d). Many papers have been retracted based on their work, and a few researchers have resigned or been removed.

The fraud-detection work of Simonsohn, Simmons, and Nelson does not give the impression that open data practices are highly effective at reducing the occurrence of researcher fraud. Making data publicly available with open data practices is hoped to reduce fraud due to the threat of detection (Stroebe et al., 2012). However, Simonsohn, Simmons, and Nelson have reported evidence of fraud in data posted publicly and in data obtained from researchers. Open data practices may increase the detection of fraud more than deter the incidence of fraud.

Various statistical methods have been proposed to screen research reports and data for possible signs of researcher fraud. As Bordewijk et al. (2021) noted in their review, these proposals currently are “rudimentary and labor-intensive” (p. 189) and have not been developed to the point of useful validation. Establishing and managing the expected error rates will be important if statistical screening methods are ever implemented.

The ORI regulations about researcher misconduct specify that relevant research records should be secured before or at the time a researcher is notified that an allegation of misconduct is being investigated. The Retraction Watch website has similarly recognized that letting researchers know about suspicions of fraud gives a fraudulent researcher an opportunity to destroy or alter records, and otherwise cover his or her tracks (McCook, 2015). In an early paper discussing data anomalies as evidence for fraud, Simonsohn (2013, p. 1886) recommended that those investigating anomalies in data “contact authors privately and transparently, and give them ample time to consider your concerns.” However, in a more recent case, they wrote a letter to the administration of the relevant university school, rather than contacting the researcher directly (Simonsohn et al., 2023a). Similarly, data anomalies discussed on the PubPeer.com website result in the responsible researcher being notified, which provides an opportunity for a fraudulent researcher to destroy or alter records. The trade-off between giving a researcher an opportunity to correct an unintentional error vs. giving a fraudulent researcher an opportunity to obscure evidence is another dilemma that has no good solution.

### 2.3 Investigation of fraud

Allegations of researcher fraud are typically investigated first by a committee from the university or research institute where the researcher is working. The committee examines all relevant records and interviews people who may have information about the conduct of research. Evidence for fraud includes data anomalies and inconsistencies that are consistent with fraud. This can include anomalies and inconsistencies in published papers, and between raw data and published data. Unambiguous deception can sometimes be identified, such as fabricating collaborators or claiming that data were collected at a certain institution, but personnel at the institution say data were not collected there.

The ORI regulations emphasize the importance of confidentiality during the investigation and “precautions to ensure that individuals responsible for carrying out any part of the research misconduct proceeding do not have unresolved personal, professional, or financial conflicts of interest with the complainant, respondent, or witnesses” [§ 93.300(b)].

### 2.4 Limitations with the retrospective strategy

#### 2.4.1 *Post-hoc* analyses

The retrospective strategy for addressing researcher fraud has the well-established weaknesses of any *post-hoc* evidence in science. Investigators have great flexibility in searching through records looking for any effects that could possibly be construed as evidence of fraud. Statistical evidence for fraud with this strategy is highly susceptible to *p*-hacking and related *post-hoc* biases that were recognized in the replication crisis in psychology (Simmons et al., 2011; Wagenmakers et al., 2012).

For example, Simonsohn et al. (2023a) reported evidence of fraud based on their finding that certain rows in a spreadsheet appeared to be out of sequence after key data values had been altered, and that these rows had results extremely favorable to the researcher's hypothesis. The researcher (Gino, 2023a) responded that the spreadsheet had other rows that had the same appearance of being out of sequence, but the data values were not favorable to her hypothesis. Gino said that Simonsohn et al. did not include in their analyses some data that were inconsistent with their speculations. She also said that the claim that rows were out of sequence was based on an incorrect understanding of the inner workings of spreadsheet software. At the time this is being written, Gino has not yet publicly responded in detail to the three other studies addressed by Simonsohn et al. (2023b,c,d). With *post-hoc* analyses, rationalizations for favorable data selections can usually be easily developed on both sides—which tends to make debates have no convincing resolution and is one reason that *post-hoc* analyses have low credibility in science.

*Post-hoc* statistical analyses may justify opening an investigation, but cannot be expected to convincingly resolve scientific debates. Evidence of fraud that is not based on *post-hoc* statistical analyses is ultimately needed for reasonably non-controversial findings.

When the weaknesses of *post-hoc* analyses are combined with the low preponderance-of-the-evidence standard for proof, this strategy has potential for incorrect outcomes (on either side), unintentional or intentional biases, and general controversy. Legal challenges will not be surprising. Gino filed a defamation lawsuit against Simonsohn, Simmons, and Nelson for over 25 million dollars (Gino v. President and Fellows of Harvard College, 2023). A crowd funding campaign for the legal defense of Simonsohn, Simmons, and Nelson has raised over $380,000 at the time of this writing (GoFundMe, 2023).

#### 2.4.2 When the person who committed fraud cannot be identified

A significant issue in this case and in some of the other cases raised by Simonsohn, Simmons, and Nelson is that even if data anomalies are interpreted as evidence of fraud, the anomalies often do not provide evidence about who committed the fraud. A study can be retracted for researcher fraud without identifying who committed the fraud.

This limitation resulted in a comment by Simonsohn in an online interview that “my belief is that she did it [committed fraud], but there is no evidence of that” (Gino v. President and Fellows of Harvard College, 2023, First Amended Complaint, August 8, 2023, paragraph 232). The lawyer for Simonsohn, Simmons, and Nelson argued that one of the reasons that defamation is not applicable is that they acknowledged that someone else could have manipulated the data (Gino v. President and Fellows of Harvard College, 2023, Memorandum in Support re 41 Motion to Dismiss for Failure to State a Claim, Nov. 8, 2023). The lawyer for Gino argued that the writings of Simonsohn, Simmons, and Nelson implied that Gino manipulated the data and did not describe the lack of evidence (Gino v. President and Fellows of Harvard College, 2023, First Amended Complaint, August 8, 2023).

An investigation conducted by the university, Harvard, concluded that Gino committed research misconduct. As part of the defamation lawsuit, the final report of the investigation (HBS Investigation Committee, 2023) was ordered released to the public, which is contrary to Harvard's usual practice. The investigation included hiring forensic consultants who compared the final data used in four studies with earlier versions of the data. These comparisons found clear discrepancies consistent with intentional data manipulation to produce the desired results.

Gino adamantly denied committing fraud and suggested that the discrepancies may have resulted from a combination of valid data cleaning and data processing steps, unintentional errors, and possible fraud by others, including someone who was attempting to damage her reputation and career. She said that research assistants collected, cleaned, and prepared the data for analyses. She also said that she allowed data, her laptop computer, and her research accounts to be accessible to coauthors, research assistants, and students. This would provide opportunities for fraud by others. The witnesses who worked with her as coauthors and research assistants testified that they trusted her completely and saw nothing that would suggest she would commit fraud. However, the investigating committee concluded by a preponderance of the evidence that fraud by Gino was more plausible than fraud by others. The committee noted that the fraud occurred at different institutions with different research assistants and different coauthors. Gino included Harvard in the defamation lawsuit.

In cases with extensive research fraud, it is surprisingly common that the person who actually committed the fraud cannot be identified (e.g., San Francisco Committee on Scientific Misconduct, 2016, 2019; ORI v. Kreipke, 2018; Van Noorden, 2022; Scientific Panel, 2023). As with Gino, data management in these cases tends to be uncontrolled with many people having access to the data and without reliable tracking of changes to the data or secure preservation of the initial raw data.

The principal investigator or laboratory director in these cases is often at the center of an extensive pattern of fraud, but manages the research in a way that prevents reasonable evidence about who did it. The research environment is highly conducive to fraud. A fraudulent or irresponsible research leader can establish research practices that preclude evidence about who committed the fraud.

The ORI regulations include a provision that the misconduct was “committed intentionally, knowingly, or recklessly” [§ 93.104(b)]. The specification that misconduct can result from reckless behavior implies that responsibility for misconduct goes beyond just the person who intentionally or knowingly performed falsification or fabrication.

ORI has a precedent that a principal investigator or laboratory director who recklessly creates a research environment conducive to fraud has responsibility for a resulting pattern of fraud when the person who intentionally performed the fraud cannot be identified (ORI v. Kreipke, 2018).

Research institutions have been inconsistent in attributing responsibility and handling fraud in these cases. In one case, the investigating committee found that several instances of fraud clearly occurred, but the person who committed the fraud could not be identified (San Francisco Committee on Scientific Misconduct, 2016). In the absence of evidence about who committed the fraud, the senior researcher accused of fraud was found to be not guilty. However, a second investigation was held for the same researcher with a similar lack of evidence about who committed the fraud. This time the finding was that the researcher “was senior/last author on all these publications and therefore responsible for the results” (San Francisco Committee on Scientific Misconduct, 2019, p. 11).

When the President of Stanford University was investigated for extensive fraud in different laboratories that he managed over the years, the investigating committee concluded that there was no evidence he personally committed the fraud and that apparently multiple unidentified people in different laboratories under his supervision committed fraud (Scientific Panel, 2023, compare with Gino above). He was found not to have committed the misconduct. His management was recognized as unusually conducive to fraud, but was described as “there may have been opportunities to improve laboratory oversight and management,” (p. 5 and 21) rather than as having responsibility for research fraud.

Similarly, an investigation of many instances of fraud in the laboratory directed by a prominent researcher concluded that there was no evidence that the laboratory director personally committed fraud (Van Noorden, 2022). However, two researchers under his supervision and training in the laboratory were found to have been responsible for fraud. They both denied responsibility for the fraud.

One of the accused researchers filed a lawsuit against the university for defamation and violation of due process rights, among other claims (Pichiorri v. Ohio State University, 2023). The ongoing lawsuit argues that the accused researcher (a) did not generate the images that were falsified, (b) was not supervisor for those who produced the images, (c) was inappropriately deemed reckless and responsible for the fraud rather than those who had relevant supervisory positions, and (d) the inadequately trained investigation committee misapplied the ORI regulations for recklessness and honest error. The lawsuit also states that the laboratory director provided no training or standards for recording and managing data.

Holding a laboratory director, principal investigator, or senior researcher accountable for a pattern of fraud that occurs under his or her supervision provides a strong incentive to implement good research practices. However, consistent application of this principle, particularly for high status scientists, will likely require a clear regulatory framework. This rationale is discussed further in comments on the proposed revisions to the ORI regulations (Kennedy, 2023c).

#### 2.4.3 The potential for deficient investigations

In an article about certain aspects of the ORI regulations, Caron et al. (2023) noted that:

the vast majority of investigation committee members lack forensic experience in assessing the conduct of their peers according to the unique regulatory framework prescribed under Part 93, which requires them to apply difficult concepts like recklessness, … “preponderance of the evidence,” and burden-shifting to the respondent to demonstrate “honest error” (p. 3).

Burden-shifting for “affirmative defenses” was emphasized in the Harvard Gino report. The proper application of this provision is a central point of dispute. Gino's lawsuit claims that the investigation committee inappropriately placed the burden of proof on Gino rather than on themselves, did not find or present any tangible evidence that Gino herself committed fraud, and inappropriately dismissed the consistent testimony from Gino's coworkers that fraud would have been out of character for her given that she did not pressure people for successful results and readily abandoned many studies that were not successful (Gino v. President and Fellows of Harvard College, 2023, First Amended Complaint, August 8, 2023). The proposed revisions to the ORI regulations (ORI, 2023, p. 69584) would remove the burden-shifting provision [ORI, 2005, § 93.106(b)(2), also see, p. 28372]. It remains to be seen whether the final regulations will retain this provision that requires legal knowledge about the differences between affirmative and negative defenses, among other points of law.

An investigation committee may also have difficulty sorting out the sometimes-conflicting roles of objective investigator, prosecutor, defender, and fact-finding decision-maker. For example, the Finding of Fact for the Gino case included that two of Gino's coauthors “were unaware of anyone besides Professor Gino having access to the data” (HBS Investigation Committee, 2023, p. 19). This appears to support the idea that Gino committed fraud. However, examination of the actual testimony reveals that both coauthors stated that they had no knowledge about who did the data cleaning and analyses for Gino's part of the research and that Gino may have delegated that to others (p. 363 and 458). In addition, a research assistant who worked with Gino on many studies said that Gino was extremely busy, was involved with developing hypotheses and study designs more than conducting the studies, “wasn't as hands-on as other professors,” “would delegate a lot,” and frequently had “many, many collaborators working in tandem” (p. 427). An alternative summary of the evidence appears to be that others besides Gino likely had access to the data and with limited oversight. The given finding of fact has the appearance of a role as prosecutor more than objective investigator.

The usual instructions for an “impartial and unbiased investigation to the maximum extent practical” (HBS Investigation Committee, 2023, p. 63) may require substantial explanation and training if the legal roles are to be properly implemented in an academic setting.

An investigation committee may be influenced by the widely-held assumption among academic scientists that fraudulent researchers are very rare. Therefore, fraud by one person may be considered more likely than fraud by multiple other people when there is no direct evidence about who committed the fraud. Also, evidence may be construed to conform to this preexisting assumption.

However, this assumption is not based on empirical evidence. Obtaining reasonably reliable data about the rarity of undetected fraud is not possible. Fraud may be much more common than many academic scientists want to believe. For example, Simonsohn et al. (2023a) reported evidence that fraud occurred independently in two different research groups (one of which was Gino) for a jointly published paper. As noted in the previous section, the possibility that multiple people committed fraud rather than one person has been considered plausible in some cases, and may be related to the status of the scientist who is the common denominator and research manager for the fraud.

Those involved in researcher fraud cases would be well-advised to ponder the possibility that attributing responsibility for fraud and construing evidence may sometimes be overly influenced by preexisting assumptions. This may be particularly applicable when scientists are out of their element in legal proceedings.

The Gino case also demonstrates that the final report and all records of the evidence in a research fraud investigation can be made public with appropriate redactions. Keeping these proceedings secret may support questionable or improper practices and reduce confidence that a case was handled adequately.

#### 2.4.4 The risks of lawsuits

Lawsuits are sometimes necessary to get the attention of a person or organization and to force them to do the right or fair thing, but lawsuits can also be counterproductive. Lawsuits can be used in attempts to intimidate people and to stifle open discussion. Also, caution is warranted in selecting lawyers and in seeking legal advice. Optimistic comments by lawyers may fit what the client wants to hear, but the client may not fully appreciate the risks.

The use of lawsuits to intimidate a person is a well-known legal tactic, particularly when one side has more resources than the other (Kassenbrock, 2023). Pursuing a defamation lawsuit to trial often takes several years and has legal fees of several hundred thousand dollars. This possibility is an untenable nightmare for a person with limited resources, even though the defendant would likely prevail if a trial was held. A lawsuit for defamation may be filed with the expectation that the defendants will settle without a trial and will publicly retract the unfavorable comments about the plaintiff.

However, defamation lawsuits can also be costly for the plaintiff. A researcher who filed two lawsuits relating to misconduct lost both lawsuits and owed over $1,000,000 in legal fees to the law firm that represented him (Kincaid, 2023). Most U.S. states now have laws to address what is known as SLAPP or *strategic lawsuit against public participation* (Kassenbrock, 2023). SLAPP lawsuits are intended to stifle public discussion and criticism. The anti-SLAPP laws can make filing a lawsuit for defamation costly to the plaintiff. For example, a scientist who filed a defamation lawsuit against those who published a paper criticizing his work dropped the lawsuit when the defendants would not settle the case—but was then ordered to pay the defendants' legal fees of over $500,000 (Oransky, 2024).

As these examples indicate, in defamation lawsuits between scientists involving scientific work, the primary winners have been the lawyers who received large legal fees whatever the outcome.

#### 2.4.5 What standard of proof is actually applied?

An investigation committee presumably is more likely to conclude that researcher fraud occurred when the evidence is strong, such as clear and convincing. In the case summaries on the ORI website, most recent cases of detected fraud have involved reuse and/or manipulation of images [ORI, (a)]. The falsification is apparent upon examination and does not require statistical analyses. As noted above, clear and convincing evidence is closer to the usual standards for scientific evidence.

Convincing evidence may occur primarily with more extreme cases of fraud. Broad and Wade (1982, p. 86) noted that most cases of detected fraud had “egregious arrogance or carelessness” by the fraudulent researcher, and “The chances of getting caught in committing a scientific fraud are probably quite small.” One of the witnesses in the investigation of Gino thought it was implausible that an experienced researcher would manipulate data in a way that was so obvious and easily detected (HBS Investigation Committee, 2023, p. 463). Ivan Oransky, co-founder of the Retraction Watch website, is quoted as saying “Most of the time, these cases don't go anywhere. The most likely outcome for someone who commits scientific misconduct is a long and successful career” (Bouffard, 2020).

### 2.5 Conclusions about the retrospective strategy

The retrospective strategy can be useful in cases of careless fraud, and particularly extensive careless fraud, but the effectiveness is questionable for less extreme cases of fraud. Strong evidence is common in cases of extensive careless fraud. However, for research based on statistical analyses, more careful fraudulent researchers, especially those with a knowledge of simulations, may be largely immune to the retrospective strategy. And, of course, AI can be expected to dramatically reduce the effort and increase the sophistication of research fraud. Science-sleuth Elisabeth Bik recently acknowledged “I think there's probably a lot of papers being produced right now that we can no longer recognize as fake” (Balthazar, 2024).

Substantial variability and inconsistency can be expected in applying the preponderance-of-the-evidence standard, in making inferences from *post-hoc* analyses, and in attributing responsibility in cases without direct evidence about who intentionally committed the fraud. Controversies and legal challenges can be expected and are very undesirable uses of scientific resources. Inadequate understanding of law by scientists combined with inadequate understanding of science by lawyers may tend to make the retrospective strategy inefficient and sometimes have unfortunate, precarious outcomes. A case with some dubious conclusions and impractical precedents by a judge who had an overly simplistic understanding of science was described in comments on the proposed changes to the ORI misconduct regulations (Kennedy, 2023c).

One useful precedent may be to hold primary investigators and laboratory directors responsible when a pattern of researcher fraud occurs under their supervision and the research practices make fraud easy, tempting, and anonymous. This accountability provides a strong incentive for primary investigators and laboratory managers to implement good data management practices that prevent fraud.

More detailed policies and training for fraud investigations could reduce the potential for inconsistency and legal misunderstandings. Providing separate findings about whether fraud occurred and about who committed the fraud would be useful. Designating findings that have clear and convincing evidence vs. findings that only meet preponderance of the evidence would also be valuable. This distinction would be useful for integrating the scientific and legal aspects of a case and for understanding the standards that were actually applied. The committee investigating researcher fraud could specify the degree of evidence for each finding. To the maximum extent possible, the full final report and record of evidence should be made publicly available, with appropriate redactions.

Those searching for evidence of fraud in published results or in data should become aware of potential legal issues, and also consider ethical standards that may not reach the threshold for legal actions. The inferences from retrospective evidence about possible fraud should be carefully described in a way that makes the limitations and uncertainties conspicuous to all readers, including journalists as well as scientists. A text box that highlights key points may be useful, including the limitations associated with *post-hoc* analyses.

Examining data for anomalies, including possible evidence of fraud, should be considered acceptable scientific practice. However, publicly expressing speculations or personal beliefs that a specific person committed fraud without direct evidence and without a thorough investigation should be recognized as unethical and unacceptable in science.

## 3 Conclusive evidence of fraud as it occurs

Conclusive evidence of fraud as it occurs is based on a sting operation that eliminates any doubts about whether fraud occurred or who did it. It also essentially eliminates any grounds for a lawsuit. Such sting operations are rare. Stroebe et al. (2012) noted only one “sting operation” in their summary of 40 cases of researcher fraud.

My experience with this type of exposé occurred when I realized that the Director of the laboratory who had hired me was fraudulently manipulating experiments (Kennedy, 2017). This was my first professional research position in the 1970's. The Director was a persuasive speaker and was held in high regard by the people whom I would have to approach with my allegations. As the newest employee of only 6 months, I had no doubt that he could easily talk his way out of the allegations and the outcome would be far worse for me than for him. I had not seen specific fraud, just his hands at some electronic equipment that could be manipulated to alter the experimental results, and very positive results during that time.

Two coworkers who had worked there longer agreed that absolutely compelling evidence was needed before any allegations were raised. We covertly set up a duplicate recording system before the point in the electronics that the Director was apparently manipulating. This provided real-time direct comparison of the original data with the manipulated data as the fraud occurred. In addition, a coworker was positioned to covertly observe the electronic equipment during the time the Director manipulated the equipment. The results were conclusive and the Director was terminated. About a year was spent investigating the extent of the fraud.

The ORI website includes sting operations in a discussion of “Set-up Experiments (S-UEs).” S-UEs include cases when a researcher is asked to repeat an experiment with careful observation as well as sting operations. ORI's conclusion about their experience with S-UEs is:

ORI concludes from these examples that set-up experiments have sometimes been problematic, especially when the members of the laboratory conducting the S-UEs have not sufficiently documented the evidence or informed institutional officials who could independently monitor or confirm the actions. However, in other cases, the S-UEs have been used successfully to confirm suspicions about research misconduct and to obtain an admission from the respondent [ORI, (b)].

As an example of a problematic case, a group of postdoctoral and graduate students thought their professor was committing fraud. The students covertly prepared an inactive agent for use by the professor in an experiment with biological samples. When the professor produced results consistent with an active agent, the students accused him of fraud. However, in the subsequent investigation, the sting operation was found to be unconvincing because the students could not prove what was in the samples they prepared. ORI also commented that the students should have established communication with the relevant authorities at the university.

All three of the sting-operations discussed by ORI were similar in that an inactive treatment was covertly introduced rather than the expected treatment. Claims about fraud were based upon the results of subsequent experiments. However, none of the cases involved direct recording and observation of fraud as it occurred. Only one of the three sting operations was successful in establishing fraud. The other two cases were ineffective and inconclusive.

### 3.1 Limitations with sting operations

Sting operations that do not achieve direct recording and/or observation of fraud as it occurs may not be effective for exposing fraud. A sting operation is vulnerable to challenge if it requires an inference about what happened without direct evidence as the fraud occurs.

Many instances of fraud are not conducive to direct observation. For example, fraud committed with a laptop computer at home cannot be reasonably observed or recorded. This substantially limits the use of sting operations.

Those considering a sting operation should prepare for the possibility that the person committing fraud will claim that he or she is being framed by people who are jealous or vindictive. This claim is likely if the person committing fraud has time to prepare a defense or has anticipated possible accusations.

Multiple people typically need to be involved in a sting operation, although this increases the possibility of leaks about the operation. Multiple people are usually needed for the intense effort to develop the logistics and the technical implementation. Also, multiple people are needed for overwhelming credibility that can neutralize counterclaims by the fraudulent researcher. When possible, those in positions of authority at the institution should be involved with the sting operation. This is particularly true when the target has a powerful position.

A sting operation aimed at a person with whom you have daily frequent interactions requires a degree of compartmentalization and gamesmanship that may be difficult for many researchers. I found it very difficult. Normal interactions must be maintained while also working intensely and covertly in making and implementing plans that will (a) ruin a colleagues' career, (b) cause an unthinkable traumatic explosion in the work environment for other colleagues, and (c) seriously damage the reputation of the institution. The stresses in this situation must be considered in deciding who to approach about becoming involved in a sting operation.

### 3.2 Conclusions about sting operations

A good sting operation is highly effective when it can be done, but may usually not be feasible. An optimal sting operation will involve duplicate records and/or direct observations that do not affect the experimental outcome if fraud does not occur. In principle, the targeted researcher need never know about the sting operation if fraud is not found.

An alternative perspective is that a sting operation is simply applying good research practices in an environment that has poor practices. Making a copy of the original data that the researchers cannot alter and independently checking and observing data collection are good research practices. The need for a sting operation implies poor research practices—as does the occurrence of fraud in general. A more effective strategy is to make the practices in a sting operation standard procedure, rather than as special secret measures used only when fraud is suspected. This would prevent the occurrence of researcher fraud, rather than respond after the fact.

## 4 Data management practices that prevent researcher fraud

The goal for practices that prevent researcher fraud is to make fraud very difficult, not easy and tempting. Practices that achieve this goal are standard procedure for clinical trials regulated by the U.S. FDA and corresponding agencies in other countries. These practices are part of an international agreement about good research practices for pharmaceutical research (International Council for Harmonization, 2024). Unfortunately, key practices appear to be largely unknown or unimplemented by academic researchers. Note that many clinical trials conducted at academic medical centers in the U.S. are not regulated by FDA and do not implement these practices.

Preventing researcher fraud also prevents the associated persistent invalid claims in the scientific literature, the waste of scientific resources, the potential for lawsuits and associated legal fees, and the inevitable trauma that occurs with allegations of fraud and sting operations. The dilemmas associated with protecting whistleblowers, giving fraudulent researchers an opportunity to cover their tracks, detecting sophisticated fraud, *post hoc* analyses, and expecting investigation committees to have a working knowledge of various unfamiliar legal points would also be minimized.

The practices that prevent fraud are valuable quality control measures that prevent unintentional errors as well as intentional errors. A research environment that is conducive to fraud is also conducive to unintentional errors. The justification for these practices in clinical trials is as much or more for unintentional errors as for fraud.

Current discussions of data management in academic research focus on sharing data and give little or no attention to practices for preventing unintentional and intentional errors (e.g., Wilkinson et al., 2016; Tenopir et al., 2020; Borghi and Van Gulick, 2021). The common academic assumption that the possibility of researcher fraud can be ignored while conducting a study appears to be reflected in data management practices—and the assumption often appears to extend to ignoring the possibility of unintentional errors as well. In some cases, this assumption may allow not only biased errors, but also error corrections that favor the researchers' hypothesis to be unconsciously applied selectively until the desired results are obtained. This possibility may seem farfetched to those without experience with research quality control. However, the possibility will likely become increasingly plausible as experience is gained with quality control.

As noted above, in cases of extensive fraud by unknown persons, the data management typically had many people with access to the data and little or no tracking of changes to the data. In response to the undocumented discrepancies in different datasets, Gino (2023b) said “there are often multiple versions of spreadsheets circulating among the RAs and principal investigators as part of the legitimate process of correcting, cleaning and consolidating data.” Gino presented this as common practice. The recent discussions of data management cited in the previous paragraph give little reason to believe that such practices are uncommon. The Gino case demonstrates why changes to the data should be carefully restricted and tracked.

The practices described below are based on guidance documents from FDA and my experience working in regulated clinical trials for about 15 years with four different organizations. Relevant FDA guidance documents cover computer systems and electronic records (U.S. FDA, 2007, 2024) and good clinical practices (U.S. FDA, 2018). I will attempt to adapt or generalize the practices to fit other types of research. Other research practices long used in clinical trials regulated by FDA have recently become widely embraced in science, including study preregistration, distinguishing between exploratory and confirmatory research, large sample sizes based on power analyses, and independent examination of the data.

The purpose here is to describe standards for practices that can be widely applied. Similar to the FDA guidance documents noted above, the standards are presented without discussing the technology and practical methods for implementing the standards. It is assumed that different research centers may use different methods for achieving the standards, and that technology will change over time. A website that could be updated frequently would be an appropriate forum for discussing the practical implementation of the standards. Some relevant points about technical matters can be found in Kennedy (2023b).

Eight topics are discussed that together provide the foundation for good data management practices that prevent researcher fraud and unintentional errors. For each topic, relevant excerpts from FDA guidance documents are given, followed by brief comments.

Reducing the intense pressure to publish in academics is a frequently-discussed alternative approach for preventing research fraud. However, that approach may be based on anecdotal evidence and speculation. Initial evidence that was more systematic did not support the hypothesis (Fanelli et al., 2015). Also, it is not clear that reducing such competition would be beneficial for science or could be realistically achieved.

### 4.1 Archive the raw data

*Ensure that the systems are designed to permit data changes in such a way that the data changes are documented and that there is no deletion of entered data (i.e., maintain an audit trail, data trail, edit trail)*. (U.S. FDA, 2018, p. 27)

*If data are transformed during processing, it should always be possible to compare the original data and observations with the processed data*. (U.S. FDA, 2018, p. 28)

The original raw data should be securely preserved to allow verification that all changes to the data were consistent with preregistered criteria for confirmatory research and with reported criteria for exploratory research. For electronic data, the easiest and most reliable way to achieve this goal in academic or non-profit research settings will usually be to place a copy of the raw data in a permanent secure repository or archive that has version control or audit trails. Users should not be able to circumvent the version control or audit trails. The best practice is to use automated data collection systems that automatically upload a copy of the data to a secure repository before the researchers have access to the data. However, a sometimes-necessary alternative may be to manually copy the data to the repository.

### 4.2 Audit trails

*Changes to source data should be traceable, should not obscure the original entry, and should be explained if necessary (e.g., via an audit trail)*. (U.S. FDA, 2018, p. 22).

*Audit trails must capture electronic record activities including all changes made to the electronic record, the individuals making the changes, the date and time of the changes, and the reasons for the changes. Original information must not be obscured by the use of audit trails or other security measures. Audit trails should be protected from modification and from being disabled*. (U.S. FDA, 2024, p. 13)

*The audit trail information should accompany all copies of the record …*

*The information should be complete and understandable with clear and concise terms to describe the components of the audit trail*. (U.S. FDA, 2024, p. 14)

All changes to the data should be tracked by some form of audit trail. Various data processing steps are typically done to create the final analysis dataset from the raw data. The data processing may be done manually and/or with programming. Although automated audit trails that cannot be circumvented are optimal, audit trails for academic research can be implemented using manually tracked logs, laboratory notebooks, or comments in computer programs.

In all cases, the audit trail should provide complete tracing and reconstruction from the final analysis data back to the initial raw data. The audit trail includes who made each change, when, and why.

### 4.3 Restricted access to data collection processes and to data

*Maintain a security system that prevents unauthorized access to the data*.

*Maintain a list of the individuals who are authorized to make data changes*. (U.S. FDA, 2018, p. 27)

Limiting who has access to the data collection process and the resulting data is a basic step in preventing research fraud. With secure archiving of a copy of the raw data and appropriate tracking of changes to the data, fraud during data collection is the only option for fraud that cannot be easily detected. Research security includes physical access to research materials and processes, as well as computer system passwords.

In a well-controlled research environment, the person responsible for data management should be able to provide a list of all the people who had change access to the data collection process and data at any given time during the study. If some of the people who may have had access to the data and data collection process cannot be identified, the data are not controlled and it will not be possible to reasonably prevent fraud or to identify who committed fraud or unintentional data errors.

### 4.4 Data collection software validation

*Ensure and document that the electronic data processing system(s) conforms to the sponsor's established requirements for completeness, accuracy, reliability, and consistent intended performance (i.e., validation)*. (U.S. FDA, 2018, p. 27).

*For purposes of this guidance, validation means a process of establishing and documenting that the specified requirements of an electronic system can be consistently fulfilled from design until decommissioning of the system or transitioning to a new system. Validation ensures that the electronic system is correctly performing its intended function*. (U.S. FDA, 2024, p. 8)

Formal software validation testing is a well-established essential step in developing software. Programming errors and oversights are inevitable for software developed by humans. Software validation is part of the price for using automated data collection systems and programs for data processing and analyses. Validation testing is done and documented by someone other than the original programmer(s). Validation is particularly important for data collection software because data problems at that stage cannot be easily corrected later. For comparison, if analysis software code has an error, that can be easily and fully corrected.

Software validation is also the first step in preventing fraud by a programmer. Proper software validation will detect programming fraud and make such fraud substantially more difficult. At a minimum, academic researchers can do the important basic step of *user acceptance testing*, in which the research staff who will be using the software do tests to verify that the software performs as expected in collecting, processing and recording data. The tests should also include efforts to break the software with unexpected keystrokes, rapid key strokes, and abruptly exiting the software at different points during the data collection process. More complete software validation may include code review and technical tests by a knowledgeable programmer.

### 4.5 Quality control: checking and double-checking

*Quality control should be applied to each stage of data handling to ensure that all data are reliable and have been processed correctly*. (U.S. FDA, 2018, p. 25)

Research fraud is much more difficult in a research environment that emphasizes quality control. Research activities by humans such as data entry, categorizing information, and computer programming are very susceptible to unintentional and intentional errors.

Clinical trials regulated by FDA have many people whose primary responsibility is checking the work of others. *Clinical research associates* (CRAs) or *monitors* visit the clinics where data are collected for a study (U.S. FDA, 2018, p. 33–37). The CRA's tasks include verifying that the clinic's own medical records match the records in the study database. These checks make data fabrication difficult, as well as detecting unintentional errors. At the data processing center for the study, *clinical data associates* (CDAs) intensely review the study data to verify that the records are complete and consistent. Multiple reviews by medical staff are also done.

These quality control efforts would not be needed if humans were perfect and never made mistakes. Many mistakes are found that justify the effort and expense of these quality control measures. For comparison, academic research rarely has a person whose primary responsibility and expertise is research quality control.

Quality control should be an accepted part of the research culture. In academic and non-profit research settings, data collection and other key steps that depend on one person who could easily make undetectable unintentional or intentional errors can at least occasionally be observed or perhaps recorded for quality control purposes. Good practice would be for the observed person to know that quality control checks will be done, but not know when. The quality control checks would be documented and could be used to compare data collected with and without quality control. Best practice would be to always have recordings or at least two people involved in critical, vulnerable research steps, with one person possibly serving as a quality control observer.

### 4.6 Blinding and preregistration

*Blinding/masking*

*A procedure in which one or more parties to the trial are kept unaware of the treatment assignment(s). Single-blinding usually refers to the subject(s) being unaware, and double-blinding usually refers to the subject(s), investigator(s), monitor, and, in some cases, data analyst(s) being unaware of the treatment assignment(s)*. (U.S. FDA, 2018, p. 4)

An optimal clinical trial is conducted double-blind. Before the study is made unblind to the personnel working on the study, the following steps will have been completed. A detailed analysis plan has been developed and submitted to FDA for review and approval. All the programming for the planned analyses has been developed, validated by another programmer, and deemed ready for use. Extensive checking and corrections of the data have been done. When the data are deemed ready for use, the database is formally *locked* (made read-only). Any subsequent change to the data requires extensive justification and authorization. After these steps have been completed, the study is unblinded and the analyses programs are run.

These procedures make researcher fraud and other research biases very difficult, including biased selective correction of errors. Complete double blinding is not always possible, but blinding is applied to the extent possible. Typically, important diagnostic procedures for a study can be done blind to the treatment group even if some of the study personnel are not blind.

Academic research is typically unblind, even when some blinding is possible. For example, if two groups are being compared, data corrections and the development of analyses programs could be done with random dummy values for the group assignment variable. In general, any randomization in a study is an opportunity for blinding.

For confirmatory research, the programming for data processing and analysis can be completed before data collection begins and then included in the study preregistration. This is an ultimate form of blinding.

### 4.7 Research audits

*Audit*

*A systematic and independent examination of trial-related activities and documents to determine whether the evaluated trial-related activities were conducted, and the data were recorded, analyzed, and accurately reported according to the protocol, sponsor's standard operating procedures (SOPs), good clinical practice (GCP), and the applicable regulatory requirement(s)*. (U.S. FDA, 2018, p. 3)

A good research audit will include directly addressing both research fraud and unintentional errors. For academic research, the high-level goals for an audit are to evaluate (a) whether good research practices were used, and (b) whether the actions and practices specified in the preregistration and/or protocol were properly implemented. As indicated in the excerpt above, written SOPs have a key role in implementing consistently high-quality research.

An audit is conducted after the research has been completed and is usually too late to make corrections to research procedures. Quality control checks during study conduct are needed to correct problems as they occur and to avoid a poor audit report.

Most academic researchers do not have experience with a good research audit that includes the possibility of fraud. Their closest experience is peer review for publication. Asking an academic researcher to conduct a research audit can be expected to result in a report that is closer to peer review than to a research audit that focuses on documented evidence that good research practices were used.

I conducted a research audit of the Transparent Psi Project (Kekecs et al., 2023) as a potential model of how my experience with audits in clinical trials could be applied in an academic setting (Kennedy, 2023a,b). Special training for auditors will be needed if audits are to be usefully implemented in academic research.

To give an idea of the tone of a research audit, when I told an FDA auditor that a programmer received electronic data from a laboratory and imported it into our standard format, the auditor asked “How do you know the programmer did not change the data?” (Kennedy, 2017, 2023a). This type of carefully worded question directly addresses both fraud and unintentional errors. I explained that a quality control person compared a random subset of the output from the programmer with another copy of the data that was not accessible to the programmer. The auditor expected tangible evidence that intentional or unintentional errors by any one person acting alone did not occur. Collusion between the programmer and quality control person was not considered.

In my experience in academic settings, a common response to the question how do you know the programmer did not change the data is that the programmer is assumed to be competent and honest. If I would have made that argument to the FDA auditor, I would have failed the audit and justifiably been fired. As noted above, the fact that programmers and others involved in research make mistakes is well-established. Ignoring that possibility creates opportunities for biased errors and biased error corrections, among other possible invalid results.

### 4.8 Data availability

*As part of an inspection, sponsors, clinical investigators, and other regulated entities may be requested to provide all records and data needed to reconstruct a clinical investigation, including associated metadata and audit trails*. (U.S. FDA, 2024, p. 7)

Widely accepted practices for open, transparent science include making publicly available a copy of the final data used in the published analyses. These data can be subjected to *post-hoc* statistical analyses looking for anomalies. As discussed above, such *post-hoc* analyses have limited value, but may justify initiating an investigation of possible researcher fraud. In practice, the data are often difficult to use because of inadequate descriptions of the data (Borghi and Van Gulick, 2021). Each data file should have an associated document file that clearly describes each variable or column, the range and meaning of the data values for each variable, the differences between similar variables, and which variables were used in the analyses.

Good research practice is to make public the raw data and audit trail records for all changes to the data, as well as the final data used in the analysis. Discrepancies with the raw data and audit trail records can provide direct evidence of fraud that does not depend on *post-hoc* statistical analyses.

The privacy of subjects is a significant issue for data that are made publicly available. Public data should have certain variables recoded, grouped, or removed to avoid revealing information that could be used to identify individual subjects.

Research that is conducted by, sponsored by, or otherwise involves for-profit organizations often has proprietary data that are not made publicly available. This can seriously limit the credibility of the research, particularly if audits or other independent verification of the data are also precluded (Ledford and Van Noorden, 2020). Some form of independent quality control verification of proprietary data should be expected.

### 4.9 Implementation notes

#### 4.9.1 Data management expertise

In clinical research regulated by FDA, data management is recognized as a profession that requires substantial expertise and specialized training. The medical experts who typically initiate and direct clinical trials usually do not claim to have expertise in data management. They defer to the data management professionals for the security of the data, including measures to prevent fraud. A study protocol is developed by a team that includes the medical experts, a statistician, data management personnel (database administrator and lead CDA), and CRAs. In academic research, data management is typically directed by a principal investigator or laboratory director who has little or no training in data management practices that prevent fraud and unintentional errors.

Academic research would be improved substantially if a more balanced team approach were used. People with technical expertise beyond the knowledge of the principal investigator can make important contributions that address both intentional and unintentional errors, and substantially improve overall research quality. Data management that is handled by specialists can also keep the investigators who have the most incentive for fraud from having opportunities to commit fraud.

Additional expertise in data management is needed in academic research, whether data management is handled by a principal investigator or by a data management professional. One or more websites could be developed to provide information about relevant software, cloud services, guidance documents, recommended standard operating procedures, and other resources pertaining to data management. An organization could be established to promote good research practices in academic science. Society for Clinical Data Management (2023) may be a useful model. Universities could offer classes with a more comprehensive, technical approach to data management. Universities could also have persons with technical expertise in data management available, similar to the way that computer programmers are usually available. Funding sources and journals could emphasize or require good data management practices, as they have done with study preregistration.

#### 4.9.2 Error-controlled data management designation

A designation or badge for *error-controlled data management* could be developed to indicate research that was conducted with the data management practices described above. As with other methodological advances like study preregistration, not all research will have these data management practices. For transparency, recognized methodological strengths and weaknesses should be clearly indicated for a research project. Badges or designations such as “preregistered” and “open data” are increasingly common. A similar badge would be appropriate for data management practices that prevent fraud and unintentional errors. A separate badge or designation could be applied for a study that has had a formal research audit.

### 4.10 Conclusions about practices that prevent researcher fraud

#### 4.10.1 Exploratory and confirmatory research

Measures to prevent researcher fraud are appropriate for both exploratory and confirmatory research. Exploratory research does not have the evidential value of confirmatory research, but is often used in seeking funding for further research. Strong incentives for fraud are present for both exploratory and confirmatory research, with substantial detrimental effects for science in both cases. Basic practices are appropriate for exploratory research, including securely archiving the raw data, audit trails, restricted access to the data and data collection processes, and validation of data collection software.

#### 4.10.2 Costs and benefits

Those who do not have experience with these practices may question whether the benefits are worth the costs. The costs and benefits of the different practices vary, and deserve individual consideration.

Some of the practices have substantial benefit with little cost, which means that not using the practices would be difficult to justify. Securely archiving copies of the raw data and maintaining at least manual audit trails of all data changes are in this category. These are the first steps for minimally adequate data management and should be expected for all scientific research. Selected quality-control checking of key research processes, particularly data collection, is another high priority that can often be done with existing staff and little added cost. Including the computer programming for data processing and analysis in the preregistration should be possible for most confirmatory research with no overall increase in effort or cost. The programming often can and should be developed with exploratory data. If data processing and analysis programming are not included in the preregistration or some data processing is done manually, the work can often be done blind with relatively little added effort.

Validation testing of data collection software requires some effort and expertise, but must be considered a necessary part of using automated data collection systems. For example, the formal validation of the data collection system for the Transparent Psi Project found a significant programming error that could have compromised the study (Kennedy, 2023a,b). The error does not indicate incompetence by the programmer. Such errors can be expected when humans develop software.

Some practices require an initial investment of time and money, but little effort and cost after the initial learning curve and software development. Providing automated unalterable audit trails is in this category. Restricted access to the data and data collection process is also in this category. Cloud services may make these practices readily available with relatively little effort.

Practices that require significant increases in expertise and potential cost include utilizing personnel trained in research quality control and research audits. These practices may not be justifiable or needed for most academic research. However, these practices are appropriate for high quality research on important or controversial topics. In general, a researcher does not need an auditor to point out that fraud by one person acting alone should not be easy and tempting, or that a good answer is needed to the question “how do you know that person X did not change the data?”

Funding sources and journals may recognize these practices as good investments and encourage or require them, as has occurred with study preregistration, open data, and large sample sizes. The costs would be included as necessary expenses in research grant proposals. The costs can be expected to decline as the practices become widely-used standard procedure.

## 5 The future of scientific fraud

This article focuses on research fraud by one person acting alone—which has been by far the most common form of researcher fraud found in the U.S. The strategies and practices for addressing fraud discussed here are probably less effective, but still usefully effective for the apparently much rarer cases of fraud that involve collusion among two or more people.

However, the future of scientific fraud also includes the rapidly growing problem of *paper mills* that approach scientific fraud as a profit-making business, with fraudulent papers for sale to unethical researchers (Else and Van Noorden, 2021; Brainard, 2023). That type of fraud involves sophisticated collusion among many people. As yet, the fraud from paper mills appears to focus on certain journals, certain publication practices, and researchers in certain countries (Van Noorden, 2023). Unfortunately, increased sophistication and more diverse researcher consumers can be expected given the potential profits and the international competition in science.

The hope that journals can develop adequate technical fraud detection methods appears overly optimistic to me. A more likely scenario is a never-ending technology race between those producing fraud and those detecting fraud. The detectors will inevitably lag behind the producers because the strategy is based on reacting rather than preventing.

My expectation is that the most effective strategy for addressing paper mills and other sophisticated fraud will be quality control and auditing professionals who verify research practices and who function independently of the researchers—similar to financial auditors. Research quality control and auditing services could be provided by government agencies, research institutions, and contract organizations. Developing this capability to identify high quality research is needed sooner rather than later.

## 6 About the author

Describing my background may be useful given that this paper goes farther in discussing both scientific methodology and legal issues than have other writings about research fraud. As noted above, my first research position included exposing the research fraud of the director who had hired me. I subsequently obtained a M.S.P.H. (Public Health) with courses focusing on environmental science and biostatistics, and also an excellent law course covering basic legal principles and terminology. My next position was working in state government for about 4 years with the primary duty of developing environmental regulations, including integrating the scientific, technical, and legal justifications. This required a thorough understanding of federal and state laws and regulations, and also included working with the Attorney General's Office to prepare legal documents for defending the legal and scientific justifications when legal challenges were made. My next position was with a non-profit environmental organization and included working with lawyers on many legal proceedings. I also coached scientists about effectively presenting scientific findings in regulatory and legal proceedings. Most scientists did not understand the differences in burden of proof between science and law, and this made them frustratingly ineffective, and sometimes counterproductive, in presenting evidence in legal settings.

After reaching a point when I no longer wished to work with or deal with lawyers, I changed careers and began doing data analysis for academic medical research. This was followed by about 15 years of work in clinical trials regulated by the U.S. FDA. The work in clinical trials further increased my knowledge of regulatory programs related to scientific research and provided experience with research methods that were much better than the common practices for academic and non-profit research.

I retired from paid work in 2011 and began working with psychology professor Caroline Watt at the University of Edinburgh in Scotland to promote improved research practices in psychology. These practices included study preregistration, distinguishing exploratory and confirmatory research, implementing meaningful power analyses, implementing measures to prevent researcher fraud, and conducting software validation (Watt and Kennedy, 2015, 2017; e.g., Kennedy, in press). These efforts began just before the replication crisis in psychology took off. In 2023, I was designated as an Honorary Research Fellow by the School of Philosophy, Psychology and Language Sciences at the University of Edinburgh.

## Author contributions

JK: Conceptualization, Writing – original draft, Writing – review & editing.
